# Comparison of acute physiological responses between one long and two short sessions of moderate-intensity training in endurance athletes

**DOI:** 10.3389/fphys.2024.1428536

**Published:** 2024-07-30

**Authors:** Rune Kjøsen Talsnes, Per-Øyvind Torvik, Knut Skovereng, Øyvind Sandbakk

**Affiliations:** ^1^ Department of Neuromedicine and Movement Science, Centre for Elite Sports Research, Norwegian University of Science and Technology, Trondheim, Norway; ^2^ Department of Sports Science and Physical Education, Nord University, Bodø, Norway

**Keywords:** cardiovascular drift, durability, endurance sport, threshold training, training characteristics, training intensity

## Abstract

**Purpose:**

To compare acute physiological responses and perceived training stress between one long and two short time- and intensity-matched sessions of moderate-intensity training in endurance athletes.

**Methods:**

Fourteen male endurance athletes (VO_2max_: 69.2 ± 4.2 mL·min^−1^·kg^−1^) performed one 6 × 10-min interval session (SINGLE) and two 3 × 10-min interval sessions interspersed with 6.5 h recovery (DOUBLE) of moderate-intensity training on two separate days, while running in the laboratory, using a counterbalanced cross-over trial. The two training days were separated into a first part/session (interval stage 1–3) and second part/session (interval stage 4–6). Respiratory variables, heart rate (HR), blood lactate concentrations (BLa), and rating of perceived exertion (RPE) were collected during sessions, whereas supine heart rate (HR) was assessed in a 60-min recovery period following sessions. Measures of perceived training stress (1–10) were assessed in the morning of the subsequent day.

**Results:**

HR, Bla, and RPE increased in the second compared to first part of SINGLE (168 ± 7 vs. 173 ± 7 bpm, 2.60 ± 0.75 vs. 3.01 ± 0.81 mmol·L^−1^, and 13.4 ± 1.0 vs. 14.8 ± 1.1-point, respectively, all *p* < 0.05). HR and Bla decreased in the second compared to first session of DOUBLE (171 ± 9 vs. 166 ± 9 bpm and 2.72 ± 0.96 vs. 2.14 ± 0.65 mmol·L^−1^, respectively, both *p* < 0.05). SINGLE revealed higher supine HR in the recovery period following sessions (65.4 ± 2.5 vs. 60.7 ± 2.5 bpm *p* < 0.05), session RPE (sRPE, 7.0 ± 1.0 vs. 6.0 ± 1.3-point, *p* = .001) and sRPE training load (929 ± 112 vs. 743 ± 98, *p* < 0.001) compared to DOUBLE. In the subsequent morning, increased levels of perceived fatigue and muscle soreness were observed following SINGLE compared to DOUBLE (7.0 ± 2.5 vs. 8.0 ± 1.0-point, *p* = .049 and 6.0 ± 2.5 vs. 7.0 ± 2.5-point, *p* = .002, respectively).

**Conclusion:**

One long moderate-intensity training session was associated with a duration-dependent “drift” in physiological responses compared to two short time- and intensity-matched sessions, thereby suggesting a higher overall training stimulus. Simultaneously, the lower cost of the two shorter sessions indicates that such organization could allow more accumulated time at this intensity. Overall, these findings serve as a starting point to better understand the pros and cons of organizing moderate-intensity training as one long versus shorter sessions performed more frequently (e.g., as “double threshold training”) in endurance athletes.

## Introduction

Endurance exercise performance is primarily limited by the athlete’s maximal oxygen uptake (VO_2max_), fractional utilization of VO_2max_ [indicated by e.g., lactate/ventilatory “thresholds” or performance oxygen uptake (VO_2_)], and work economy/efficiency (Joyner and Coyle, 2008). To provide a stimulus for improving endurance performance, a sufficient training load must be achieved through the interaction between training volume, intensity, and frequency. Retrospective analyses of elite to world-class endurance athletes have reported annual training volumes ranging from 500 to 1,200 h depending on the sport-specific demands, with most training performed as low intensity (∼70–90%), supplemented by 10%–30% as moderate- to high-intensity training (Seiler, 2010; Stöggl and Sperlich, 2015; Sperlich et al., 2023; Staff et al., 2023). Although most scientific literature emphasizes the effects and underlying mechanisms of high-intensity training (Laursen and Jenkins, 2002; Laursen, 2010; Stöggl and Sperlich, 2015), endurance athletes, and particularly elite endurance athletes, perform surprisingly small volumes of high-intensity training and often substantially larger volumes of both low- and moderate-intensity training (Burnley et al., 2022; Haugen et al., 2022; Casado et al., 2023; Sandbakk et al., 2023).

Moderate-intensity training is performed between the first and second lactate/ventilatory “threshold” and therefore often referred to as “threshold training” (Seiler, 2010). This type of training can be performed both as continuous sessions and as intervals with relatively long work duration. In this context, an increasingly popular method adopted across different endurance sports is so-called “double-threshold training,” which means that two “threshold sessions” are performed on the same day (Bakken, 2022; Casado et al., 2023), with “easy days” of low-intensity training in between. Although there is limited scientific literature to support the use of “double-threshold training,” information from sports practice indicates that the method originates from, and is currently well established in middle- and long-distance running (Tjelta, 2019; Casado et al., 2023; Kelemen et al., 2023). Although the volume of each session is often reduced compared to performing one longer session, the aim is normally to increase the accumulated time at relatively high, competition-specific intensities. The internal exercise intensity during such sessions, is typically around 82%–87% of maximal heart rate (HR_max_), 2–4 mmol·L^−1^ in blood lactate concentrations (Bla), and 12–16 in rating of perceived exertion (RPE) using the 6–20-point Borg scale (Seiler, 2010; Bakken, 2022; Casado et al., 2023).

Previous studies have speculated that higher overall volume of moderate-intensity training effectively drives positive adaptations related to the primary performance-determining factors in middle- and long-distance running (Bakken, 2022; Kelemen et al., 2023). In addition, lower injury risk through reduced mechanical loading combined with lower metabolic and autonomic disturbance per session (i.e., reduced recovery time) could be beneficial when performing two shorter sessions compared to one longer session (Bakken, 2022; Casado et al., 2023). However, another approach might be to split a long moderate-intensity session into two shorter sessions to allow a compensatory higher speed or power output and, thereby obtain higher external intensity/load at the same internal intensity/load [i.e., Bla, RPE, and heart rate (HR)] (Bakken, 2022; Casado et al., 2023). Although different approaches and potential benefits of performing two shorter sessions compared to one longer moderate-intensity session may exist, further examination is required to elucidate the underlying mechanisms.

In contrast, the benefits of performing fewer, but longer sessions at moderate intensity may include a larger acute training stimulus caused by greater work per session and a duration-dependent “drift” in internal intensity measures, which is speculated to upregulate molecular signaling and subsequent adaptations (Seiler, 2010; Maunder et al., 2021). Such changes in internal intensity-measures have previously been observed during prolonged sessions of low intensity endurance training in well-trained cyclists (Rønnestad et al., 2011; Hopker et al., 2017). It has also been shown that “drift” in internal intensity measures associated with performing one long compared to two shorter distance-matched sessions at low-intensity in national-level cross-country skiers influences subsequent measures of perceived training stress (Talsnes et al., 2022). However, the question is probably not whether “double-threshold training” is superior to one single session or vice versa, but rather to understand their different signal-to-stress ratios and choose the best tool at the right time for the purpose of maximizing adaptations and performance development. Taken together, despite information from sports practice emphasizing beneficial “effects” of adopting “double-threshold training” in elite to world-class endurance athletes (Bakken, 2022; Casado et al., 2023), the method has received little attention in the scientific literature. While training intervention studies are evidently required to investigate the actual training effects, descriptive studies on acute physiological responses at the same external intensity might be a starting point to better understand load and recovery differences between these types of organizing moderate-intensity (“threshold”) training.

Therefore, the present study compared acute physiological responses and perceived training stress between one long and two short time- and intensity-matched sessions of moderate-intensity training in endurance athletes. It was hypothesized that performing one long session would induce higher overall physiological responses and perceived training stress compared to two time- and intensity-matched short sessions.

## Methods

### Participants

Fourteen trained male endurance athletes (national-level cross-country skiers, n = 11 and runners, n = 3) volunteered to take part in the study. Physiological and anthropometrical characteristics of the group are presented in Table 1. The inclusion criteria specified that participants had to be between 18–35 years of age, that they perform at least 5 endurance training sessions per week, with more than 5 years experience of endurance training and without any major interruption due to injury or illness. The participants were not familiar with performing “double-threshold training” prior to the study although all participants were familiar with running in relatively steep uphill’s. The Regional Committee for Medical and Health Research Ethics waives the requirement for ethical approval for such studies. Therefore, the study was approved by the Norwegian Centre for Research Data and conducted in accordance with the institutional requirements and the Declaration of Helsinki. All participants gave their oral and written consent before participation.

**TABLE 1:** Anthropometrical characteristics and physiological tests of the fourteen male endurance athletes participating in the study.

Age (y)	23.1 ± 4.1
Body height (cm)	176.7 ± 4.2
Body mass (kg)	71.5 ± 8.3
Body mass index (kg·m^−2^)	22.9 ± 1.9
Incremental test to exhaustion	
VO_2max_ (mL·min^−1^)	4,896 ± 495
VO_2max_ (mL·min^−1^·kg^−1^)	69.2 ± 4.2
HR_max_ (bpm)	196 ± 6
Blood lactate profile test (4 mmol**·**L^−1^)	
Speed at 4 mmol·L^−1^ (km·h^−1^)	10.7 ± 0.7
Speed at 90% of 4 mmol·L^−1^ (km·h^−1^)	9.6 ± 0.5
VO_2_ at 4 mmol·L^−1^ (mL·min^−1^)	3,983 ± 511
VO_2_ in % of VO_2max_ at 4 mmol·L^−1^	82.1 ± 4.2

VO_2max_, maximal oxygen consumption; HR_max_, maximal heart rate; bpm, beats per minute; VO_2_, oxygen uptake.

### Design

The study was a counterbalanced cross-over trial in which the participants reported to the laboratory on three separate occasions. Initially, preliminary physiological testing was performed within 2 weeks of the experimental trial as a part of the participants regular testing regimes. A preliminary test, including submaximal and maximal stages, was used to determine individual workloads for the subsequent moderate-intensity training sessions constituting the experimental trial. The experiment included two training days matched for the same time and external intensity consisting of either one 6 × 10-min interval session (SINGLE) or two 3 × 10-min interval sessions interspersed by 6.5 h of recovery in between (DOUBLE), conducted while running in the laboratory. In all analyses, SINGLE was separated into a first part (interval stage 1–3) and second part (interval stage 4–6), and DOUBLE separated into two sessions [first session (interval stage 1–3) and second session (interval stage 1–3)]. Respiratory variables, HR, Bla, blood glucose concentrations (BG), and RPE were collected during each session, whereas supine HR was assessed during a 60-min recovery period following each session. Different measures of perceived training stress and recovery were assessed both 15 min following each session and in the morning of the subsequent training day.

### Preliminary physiological testing

The participants performed a blood lactate profile test followed by an incremental test to exhaustion, running at a 10.5% fixed incline using protocols previously described (Talsnes et al., 2021). The blood lactate profile test consisted of 5-min stages with increasing speeds (1 km·h^−1^) until the participants reached a Bla value of >4 mmol·L^−1^. Speed at 4 mmol·L^−1^ was calculated using linear interpolation The subsequent incremental test to exhaustion included increasing speeds by 1 km·h^−1^ every min until exhaustion, with a plate in VO_2_ despite of increasing speed as the main criteria for achieving VO_2max_ (See Table 1 for data from the preliminary physiological test).

### Experimental trial

**FIGURE 1:**
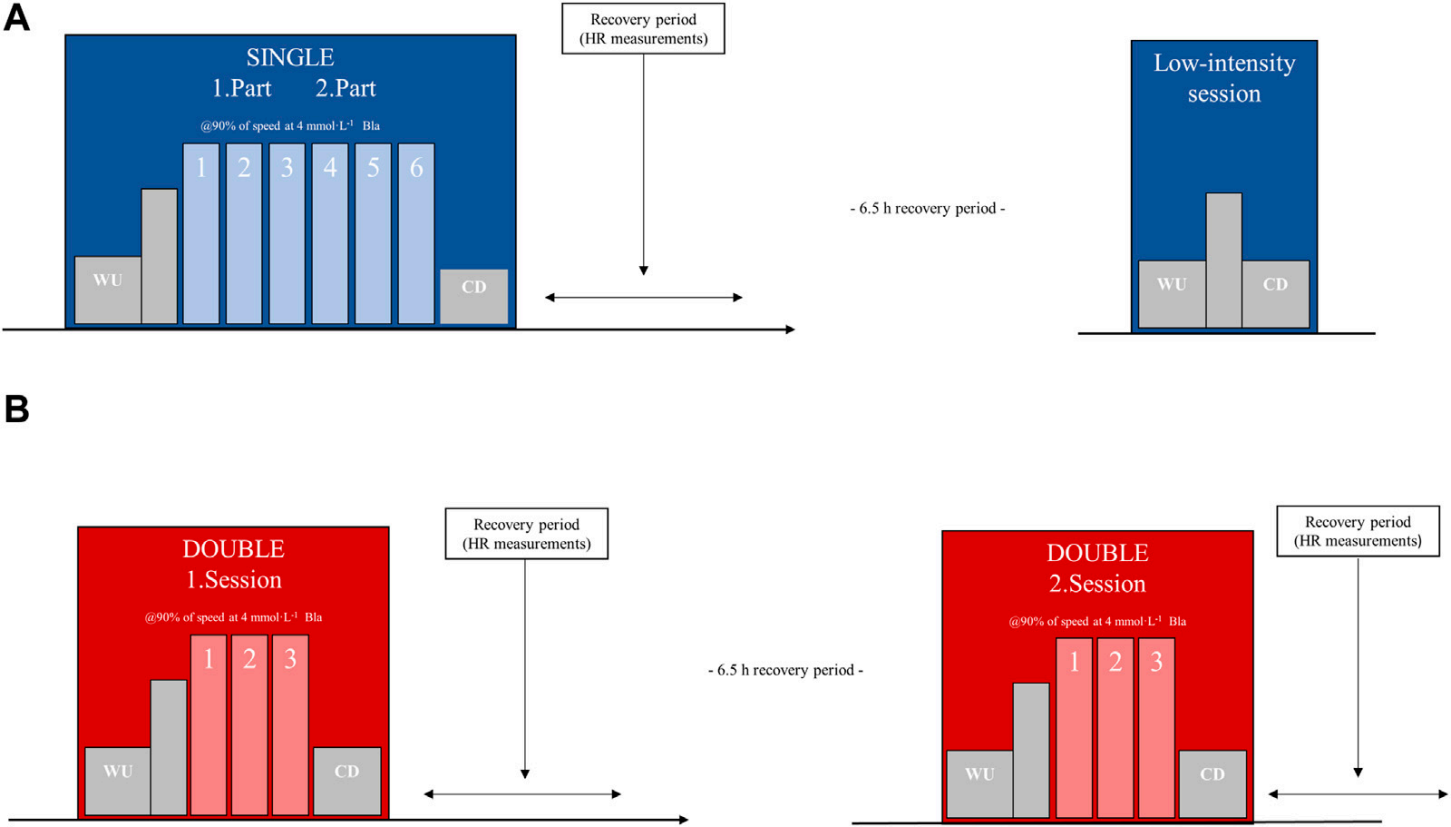
Complete protocol of the experimental trial consisting of **(A)** one 6 × 10-min interval session (SINGLE) and **(B)** two 3 × 10-min interval sessions interspersed with 6.5 h recovery (DOUBLE) of moderate-intensity training on two separate days, while running in the laboratory, using a counterbalanced cross-over trial. WU, warm-up; CD, cool-down; Bla, blood lactate concentrations; HR, heart rate.

The rationale for the experimental design and different sessions constituting SINGLE and DOUBLE were based on combination of verbal communication with coaches of elite- to world-class endurance athletes regularly performing “double-threshold training” and available scientific literature (Bakken, 2022; Casado et al., 2023) The experimental trial involved all participants, with half of them commencing with SINGLE and the other half with DOUBLE on the first training day, respectively. The complete study protocol is shown in Figure 1. Both training days were time- and intensity-matched and conducted at the same external intensity, corresponding to 90% of the participants’ speed at 4 mmol·L^−1^ from the preliminary blood lactate profile test. Based on extensive experience with physiological testing and verbal communication with sports practice, this external intensity was considered appropriate to elicit internal intensity measures that aligns with the use of “threshold training”. SINGLE comprised one 6 × 10-min interval session, while DOUBLE consisted of two 3 × 10-min interval sessions interspersed with 6.5 h of recovery in between. The recovery time between each interval stage during sessions was set to 2 min. Before each session, participants underwent a 20-min standardized warm-up protocol, including 15 min at a fixed incline and speed (5% and 8 km·h^−1^), followed by 5 min at the same incline as the intervals and a fixed speed (10.5% and 8 km·h^−1^). Following completion of the intervals, participants underwent a standardized cool-down protocol comprising 15 min at a fixed incline and speed (5% and 8 km·h^−1^). The relatively steep uphill was chosen for the purpose of the study in attempt to isolate physiological responses and decouple potential differences in running technique between participants. To time- and intensity-match the volume of both low- and moderate-intensity training across the two training days, a short low-intensity session (warm-up and cool-down protocol) was performed 6.5 h after SINGLE due to the additional warm-up and cool-down performed in connection with the second session of DOUBLE. The experimental trial was conducted in the laboratory under steady room temperature (17°C–19°C) and humidity (35%–45%). Participants were instructed to engage in low-intensity training exclusively during the last 2 days before the experimental trial and to replicate the same training regimen before both training days. The participants’ average training volume (exclusively low-intensity training) over the last 2 days was 2.0 ± 0.9 h and 1.9 ± 0.8 h before the SINGLE and DOUBLE sessions, respectively.

### Nutritional protocol

In best attempt to standardize nutritional status, the participants were instructed to replicate their dietary intake both the day before and the day constituting the experimental trial. During sessions, the participants consumed sports drink (Maxim, Orkla, Oslo, Norway) with the total fluid and carbohydrate (CHO) intake matched (1 L·h^−1^) between the two training days. The amount of sports drink and CHO intake (70 g·h^−1^) were according to ACSM guidelines on recommended CHO intake during endurance exercise (Rodriguez et al., 2009). In the 60-min recovery period following each session, the participants consumed a 185-kcal energy bar (New Energy, Nidar, Orkla, Trondheim, Norway) and a 100-kcal banana (BAMA Gruppen, Oslo, Norway) after 15 min, whereas water ad libitum were provided during the entire recovery period.

### Physiological responses

Respiratory variables including VO_2_, minute ventilation (VE), and respiratory exchange ratio (RER) were collected over the entire 10-min interval stages. HR was monitored continuously during sessions and in the subsequent 60-min recovery period. Bla, BG, and RPE were collected after each interval stage was completed.

### Supine heart rate

As a measure of autonomic recovery, supine HR was quantified 15 min before and 15, 30, 45 and 60 min following each session during a 5-min period where the participants lied down on a gym mat in the laboratory. The participants average HR over the last minute of the 5-min period was used for analyses.

### Measures of perceived training stress and recovery

Prior to each session, the participants reported their perceived “motivation” and “readiness” on a scale ranging from 1 (poor) to 10 (excellent). 15-min following each session, the participants gave their session RPE (sRPE) on a 1–10 scale. Internal training load was calculated by multiplying the participants sRPE by the duration of each session in minutes (Halson, 2014). The participants further gave their perceived training quality on a scale ranging from 1 (poor) to 10 (excellent) from a physical, technical, and mental perspective using the training quality scale recently developed by Shell et al. (2023). Lastly, measures of perceived training stress and recovery were reported in the morning of the subsequent training day including questions on sleep quality, general mental and physical wellbeing, readiness to train, muscle soreness, fatigue, and attractiveness to the training day from 1 (poor) to 10 (excellent) (Ten Haaf et al., 2017).

### Equipment and materials

The experimental trial was performed on a 2.5 × 0.7-m treadmill (RL 3500E, Rodby, Vänge, Sweden). Respiratory variables were collected using open-circuit indirect calorimetry with mixing chamber (Vyntus CPX, CareFusion, Hoechberg, Germany). Bla and BG were taken from the fingertip of the participants and analyzed using the stationary Biosen C-Line lactate device (Biosen, EKF Industrial Electronics, Magdeburg, Germany). HR was recorded using a Garmin Forerunner 935 watch with electrode belt (Garmin Ltd., Olathe, KS, United States). RPE was determined using the 6–20-point Borg scale (Borg, 1970). The participants body masses and heights were measured using a medical weight and stadiometer (Seca model 708, Seca GmbH, Hamburg, Germany).

### Statistical analyses

Data are reported as mean ± standard deviation (SD) for continuous variables and median ± interquartile range (IQR) for ordinal variables. Normality was checked using a combination of histograms and QQ-plots. A mixed linear model was used for analyses, specifically using the “lme4” package in R version 4.2.2 (R Development Core Team, Vienna, Austria). The model aimed to compare physiological responses between the two training days of different moderate-intensity training organization. The model included fixed effects for training day (SINGLE vs. DOUBLE) and part/session (first part/session vs. Second part/session) within the different training days. Additionally, the model included interactions between the fixed effects and a random effect specified for the participants variability. Where fixed effects were evident, Tukey post hoc comparisons were performed to assess specific differences. Further, measures of perceived training stress and recovery between SINGLE and DOUBLE were compared using the non-parametric Wilcoxon signed-rank test. Hedges *g* effect sizes were also calculated (Lakens, 2013) and interpreted as: 0.2–0.5 = small effect, 0.5–0.8 = moderate effect, and >0.8 large effect (Hopkins et al., 2009). The significance level for all comparisons was set at alpha levels of *p* < 0.05.

## Results

### Acute physiological responses

**FIGURE 2:**
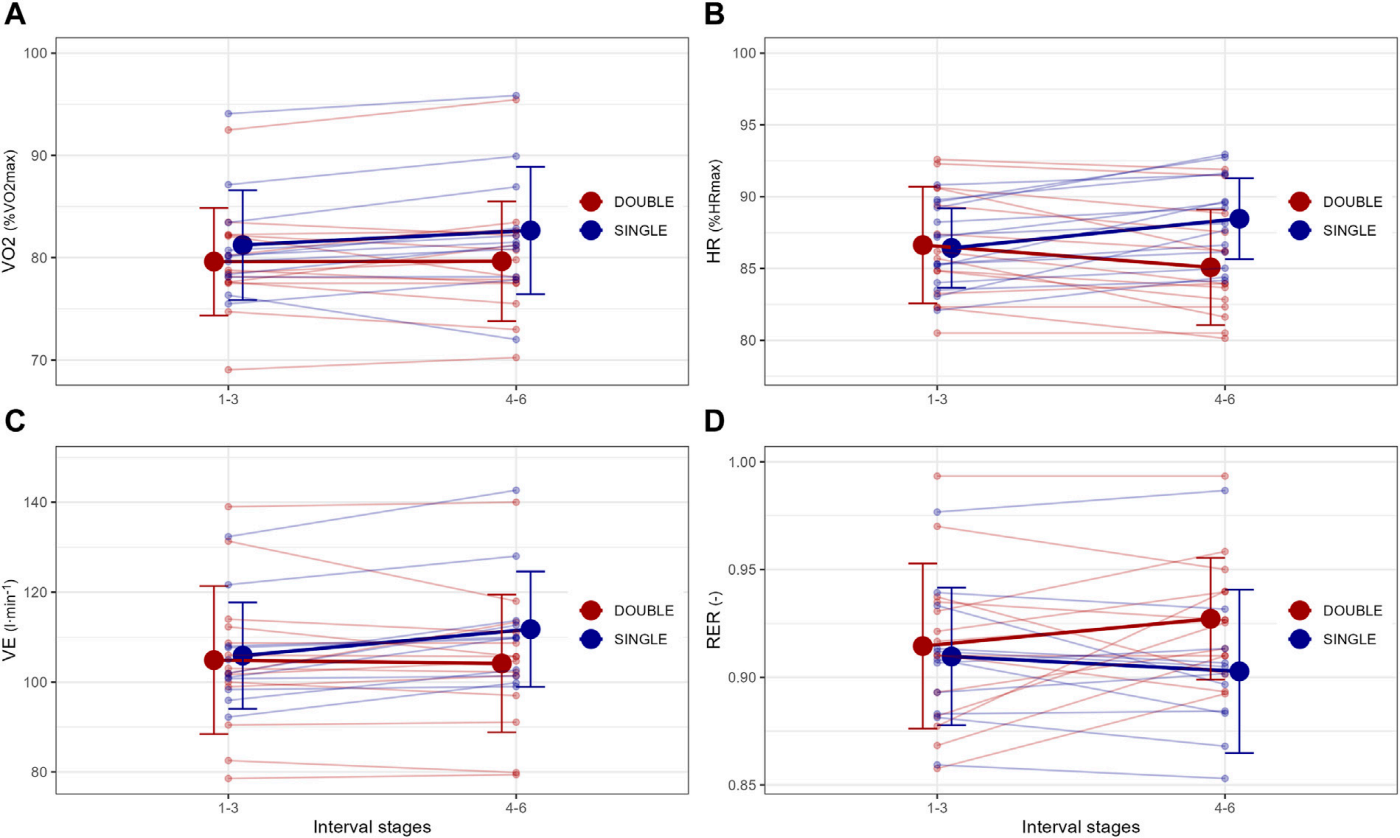
Acute responses in **(A)** oxygen uptake in percentage of maximal oxygen uptake (VO_2max_), **(B)** heart rate in percentage of maximal heart rate (HR_max_), **(C)** minute ventilation (VE), and **(D)** respiratory exchange ratio (RER) to different organization of time- and intensity-matched moderate-intensity training in fourteen male endurance athletes.

**FIGURE 3:**
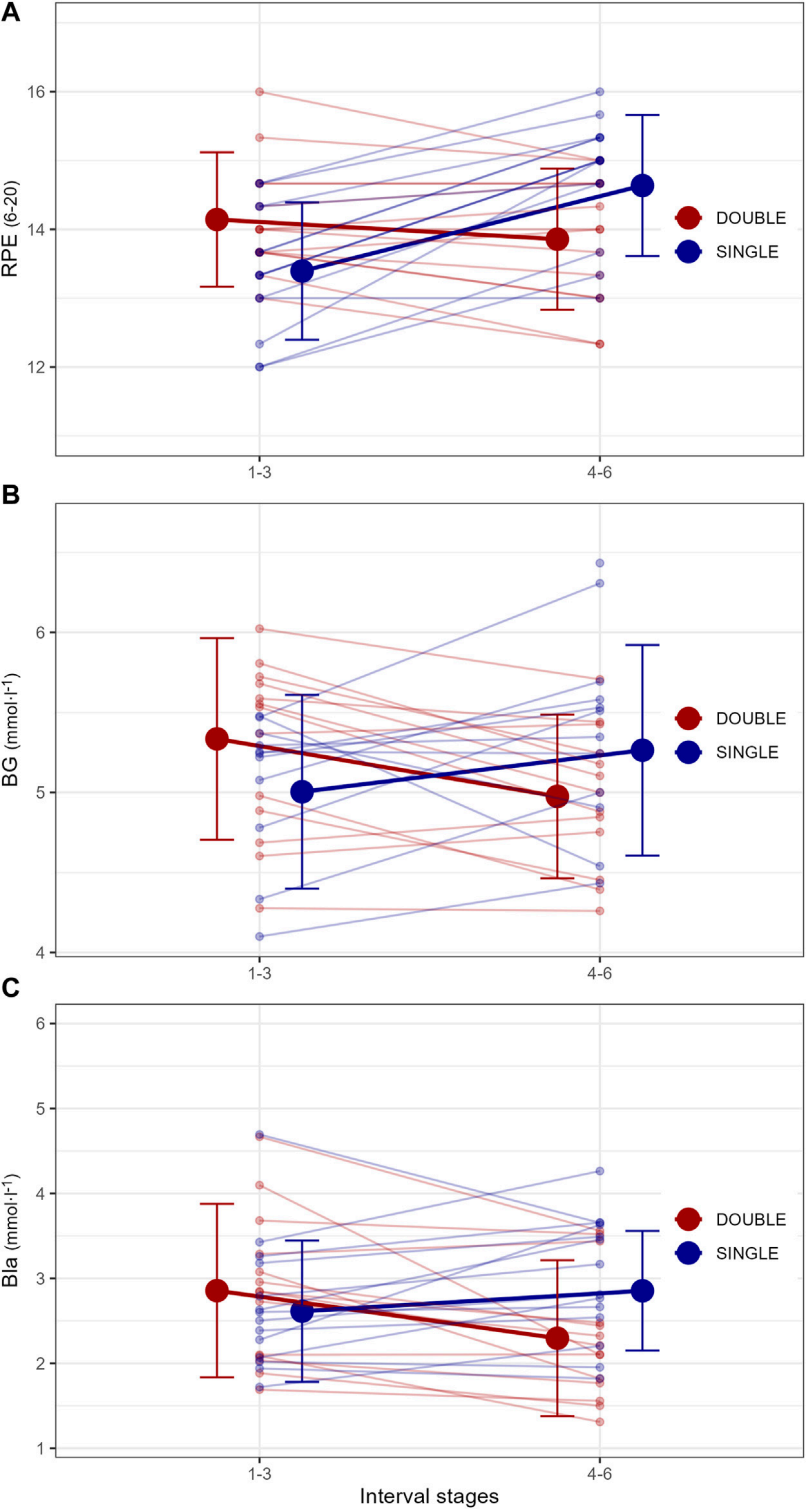
Acute responses in **(A)** rating of perceived exertion (RPE), **(B)** blood glucose concentrations (BG), and **(C)** blood lactate concentrations (Bla) to different organization of time- and intensity-matched moderate-intensity training in fourteen male endurance athletes.

Data on acute physiological responses between moderate-intensity sessions are presented in Table 2 and Figures 2, 3. There was a 2.8% ± 1.8% higher HR (average across the different interval stages) in the second vs. First part of SINGLE, and −2.5% ± 2.3% lower HR in the second vs. First session of DOUBLE (all *p* < 0.05). Further, there was an interaction effect revealing 4.2% ± 2.8% higher HR in the second part of SINGLE vs. Second session of DOUBLE (all *p* < 0.001).

**TABLE 2:** Acute physiological and perceptual responses to different organization of time- and intensity-matched moderate-intensity training in fourteen male endurance athletes.

	SINGLE		DOUBLE		SINGLE vs. DOUBLE	
	First part	Second part	First session	Second session	First part/session	Second part/session
Interval stage 1 (4)					ES (Hedges g)	
HR (bpm)	167 ± 8	173 ± 7*	167 ± 9	166 ± 8^#^	0.00	0.90
HR in %HR_max_	85.1 ± 2.8	88.3 ± 3.0	85.1 ± 3.8	84.6 ± 2.8	0.00	1.19
VO_2_ (mL·min^−1^)	3,962 ± 433	4,078 ± 488	3,840 ± 500	3,829 ± 488	0.26	0.49
VE (L·min^−1^)	102 ± 12	111 ± 14*	101 ± 15	101 ± 14^#^	0.07	0.68
RER	0.92 ± 0.03	0.91 ± 0.04	0.92 ± 0.04	0.93 ± 0.03^#^	0.00	0.55
Bla (mmol·L^−1^)	2.49 ± 0.74	2.98 ± 0.82*	2.64 ± 0.94	2.17 ± 0.66*^, #^	0.16	1.04
BG (mmol·L^−1^)	4.78 ± 0.54	5.51 ± 0.63*	5.02 ± 0.65	4.84 ± 0.47^#^	0.39	0.85
RPE (6–20)	13.1 ± 0.8	14.5 ± 1.2*	13.6 ± 0.8	13.4 ± 0.8^#^	0.58	1.02
Interval stage 2 (5)						
HR (bpm)	170 ± 7	173 ± 7*	170 ± 9	165 ± 9*^, #^	0.00	0.96
HR in %HR_max_	86.6 ± 2.8	88.4 ± 3.0*	86.9 ± 3.9	84.4 ± 4.3*^, #^	0.08	1.04
VO_2_ (mL·min^−1^)	4,031 ± 418	4,077 ± 443	3,908 ± 500	3,911 ± 508	0.26	0.33
VE (L·min^−1^)	106 ± 14	112 ± 14*	106 ± 18	104 ± 15^#^	0.00	0.52
RER	0.91 ± 0.03	0.90 ± 0.04	0.91 ± 0.04	0.92 ± 0.03^#^	0.00	0.50
Bla (mmol·L^−1^)	2.72 ± 0.82	3.03 ± 0.80*	2.80 ± 0.97	2.10 ± 0.63*^, #^	0.08	1.25
BG (mmol·L^−1^)	5.08 ± 0.40	5.53 ± 0.62*	5.45 ± 0.56	4.91 ± 0.39*^, #^	0.74	1.17
RPE (6–20)	13.3 ± 1.2	14.7 ± 0.9*	14.2 ± 1.0^#^	13.9 ± 1.0^#^	0.78	0.79
Interval stage 3 (6)						
HR (bpm)	171 ± 7	174 ± 7	172 ± 9	168 ± 9*^, #^	0.12	0.72
HR in %HR_max_	87.5 ± 2.8	88.7 ± 2.9	87.6 ± 4.0	85.9 ± 4.4*^, #^	0.01	0.45
VO_2_ (mL·min^−1^)	4,052 ± 442	4,096 ± 464	3,947 ± 493	3,956 ± 510	0.21	0.26
VE (L·min^−1^)	109 ± 14	112 ± 14	108 ± 17	107 ± 17	0.06	0.30
RER	0.90 ± 0.04	0.90 ± 0.04	0.91 ± 0.04	0.92 ± 0.03^#^	0.22	0.48
Bla (mmol·L^−1^)	2.83 ± 0.93	2.90 ± 0.73	3.13 ± 1.23	2.62 ± 1.29	0.30	0.22
BG (mmol·L^−1^)	5.24 ± 0.76	5.32 ± 0.73	5.52 ± 0.61	5.17 ± 0.63	0.23	0.20
RPE (6–20)	13.8 ± 1.0	15.2 ± 0.9*	14.6 ± 1.0^#^	14.2 ± 1.2^#^	0.77	0.90

SINGLE, one 6 × 10-min “threshold interval session”; DOUBLE, two 3 × 10-min “threshold interval sessions”; ES, effect size; HR, heart rate; HR_max_, maximal heart rate; VO_2_, oxygen consumption; VE, minute ventilation; RER, respiratory exchange ratio; Bla, blood lactate concentrations; BG, blood glucose concentrations; RPE, rating of perceived exertion.

*Significant different from first part/session within SINGLE, and DOUBLE (*p* < 0.05).

^#^Significant different from SINGLE (*p* < 0.05).

There were no significant differences either between or within SINGLE and DOUBLE in VO_2_ although there was a 6.7% ± 3.5% higher VE (average across the different interval stages) in the second vs. First part of SINGLE. Further, there was an interaction effect revealing 4.9% ± 3.9% higher VE in the second part of SINGLE vs. Second session of DOUBLE (all *p* < 0.001). There was also an interaction effect revealing 0.025 ± 0.020 lower RER in the second part of SINGLE vs. Second session of DOUBLE (all *p* < 0.05).

There were 0.46 ± 0.50 mmol·L^−1^ and 0.54 ± 0.70 mmol·L^−1^ higher Bla and BG (average across the different interval stages) in the second vs. First part of SINGLE, as well as −0.59 ± 0.65 mmol·L^−1^ and −0.32 ± 0.44 mmol·L^−1^ lower Bla and BG in the second vs. First session of DOUBLE (all *p* < 0.05). Further, there were interaction effects demonstrating 0.91 ± 0.88 mmol·L^−1^ and 0.46 mmol·L^−1^ higher Bla and BG in the second part of SINGLE vs. Second session of DOUBLE (all *p* < 0.001). Lastly, there was a 1.4 ± 0.8-point higher RPE (average across the different interval stages) in the second vs. First part of SINGLE (all *p* < 0.01), as well as an interaction effect revealing 1.0 ± 0.7-point higher RPE in the second part of SINGLE vs. Second session of DOUBLE (all *p* < 0.05).

### Supine heart rate

**FIGURE 4:**
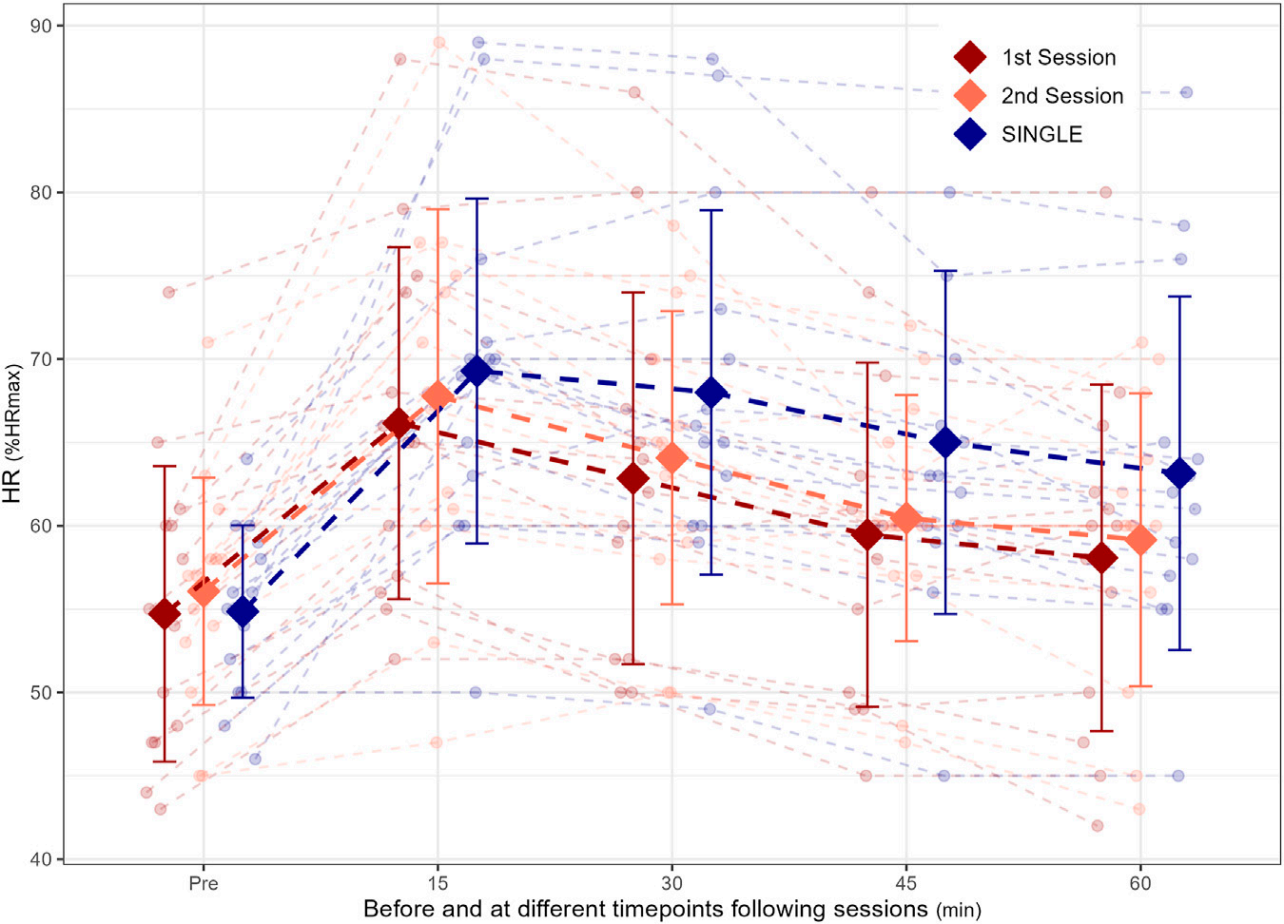
Supine heart rate (HR) before and at four time points following different organization of time- and intensity-matched moderate-intensity training in fourteen male endurance athletes.

Supine HR responses in the subsequent recovery period were 7.8% ± 12.3%, 9.4% ± 13.4%, and 9.0% ± 13.7% higher 30, 45, and 60 min following SINGLE, respectively, compared to the average values of the two sessions constituting DOUBLE, (all *p* < 0.05, Figure 4). There were no significant differences between the first vs. Second session of DOUBLE.

### Measures of perceived training stress and recovery

There was a −1.0 ± 0.5-point lower motivation in the second vs. First session of DOUBLE (*p* = .041, Table 3), with no other differences in perceived motivation and readiness before sessions reported. Further, no differences between SINGLE and DOUBLE in perceived training quality following sessions were reported, while higher sRPE and sRPE training load were evident for SINGLE compared to DOUBLE (−1.0 ± 0.7-point, *p* = .001 and 19.6% ± 9.3%, *p* < 0.001, respectively). In the morning of the subsequent training day, increased levels of perceived fatigue and muscle soreness were reported following SINGLE compared to DOUBLE (−1.0 ± 1.5, *p* = .049 and −1.0 ± 1.5, *p* = .002, respectively, Table 3).

**TABLE 3:** Perceived training stress and recovery between two different organizations of time- and intensity-matched moderate-intensity training in fourteen male endurance athletes.

	SINGLE	DOUBLE		
	Total	First session	Second session	Total
Variables pre				
Motivation (1–10)	9.0 ± 2.0	9.0 ± 2.5	8.0 ± 1.8*	8.5 ± 2.3
Readiness (1–10)	7.5 ± 1.0	8.0 ± 1.0	7.5 ± 1.8	7.5 ± 1.0
Variables Post				
sRPE (1–10)	7.0 ± 1.0	6.0 ± 0.8	6.0 ± 1.3	6.0 ± 1.3^#^
sRPE load	929 ± 112	371 ± 47	371 ± 59	743 ± 98^#^
Physical training quality (1–10)	8.0 ± 1.5	8.0 ± 1.8	7.5 ± 1.0	7.8 ± 1.4
Technical training quality (1–10)	8.0 ± 1.8	7.0 ± 1.0	7.5 ± 1.8	7.5 ± 1.5
Mental training quality (1–10)	8.0 ± 1.0	8.0 ± 1.8	7.0 ± 2.5	7.5 ± 1.9
	SINGLE		DOUBLE	
Variables				
Sleep quality (1–10)	8.0 ± 1.0		7.5 ± 1.0	
General mental wellbeing (1–10)	8.0 ± 1.0		9.0 ± 1.0	
General physical wellbeing (1–10)	8.0 ± 1.7		8.0 ± 1.7	
Readiness to train (1–10)	7.0 ± 1.5		7.5 ± 1.0	
Muscle soreness (1–10)	6.0 ± 2.5		7.0 ± 2.5^#^	
Fatigue (1–10)	7.0 ± 2.5		8.0 ± 1.0^#^	
Attractiveness to the training day (1–10)	7.5 ± 1.0		8.0 ± 1.0	

SINGLE, one 6 × 10-min “threshold interval session”; DOUBLE, two 3 × 10-min “threshold interval sessions”; Pre, 15-min before sessions, Post, 15-min after sessions; sRPE, session rating of perceived exertion.

*Significant different from first session within DOUBLE (*p* < 0.05).

^#^Significant different from SINGLE (*p* < 0.05).

## Discussion

The present study compared acute physiological responses and perceived training stress between one long and two short time- and intensity-matched sessions of moderate-intensity training in endurance athletes. In accordance with our hypotheses, performing one long session was associated with overall higher physiological responses, attributed to a duration-dependent “drift” in HR, Bla, and RPE during the second compared to first part of the long session. Conversely, reductions in HR and Bla were observed in the second compared to first session during the performance of two short sessions. Additionally, performing one long session led to higher supine HR during a 60-min recovery period following sessions, as well as elevated session RPE and consequently training load, in comparison to two short sessions. Lastly, higher levels of perceived fatigue and muscle soreness were evident the following morning after the long session compared to the two short sessions.

### Acute physiological responses

This study represents the first attempt to compare acute physiological responses between different methods of organizing moderate-intensity endurance training, specifically contrasting one long session with the increasingly popular “double-threshold training” approach, also by some, referred to as “the Norwegian method”. As anticipated, engaging in one long session induced a duration-dependent “drift” in several internal intensity measures, resulting in significant overall higher acute physiological and perceptual responses compared to two shorter sessions. These significant physiological responses (e.g., HR and RPE) between one long and two shorter sessions were further strengthened by the large effect sizes revealed for most interval stages, implying practically relevant differences. The observed changes in the ratio between internal-to-external intensity measures during the long session are consistent with the recent concept of physiological “durability/resilience,” characterized by the deterioration of physiological measures over time during prolonged endurance exercise (Maunder et al., 2021). While VO_2_ and energy cost remained relatively stable both between and within the two training days, the increased HR during SINGLE likely stemmed from cardiovascular “drift,” a well-recognized phenomenon in internal intensity measures during prolonged endurance exercise (Maunder et al., 2021; Smyth et al., 2022). This phenomenon is thought to be caused by decreased stroke volume and increased sympathetic nervous activity (Souissi et al., 2021). The observed increase in VE aligns with previous findings during prolonged endurance exercise, although in prior studies, this has been coupled with a “drift” in VO_2_ and energy cost (Rønnestad et al., 2011; Hopker et al., 2017), which were not evident in the present study. This discrepancy may be attributed to the shorter duration and higher exercise intensity in our study compared to previous investigations of physiological “drift” during more prolonged low-intensity sessions (Rønnestad et al., 2011; Hopker et al., 2017). Moreover, Bla and BG concentrations increased from the first to second part of SINGLE, likely reflecting increased glycolytic energy turnover as the session progressed. These findings are consistent with previous studies of physiological responses to prolonged low-intensity training and are likely attributable to increased recruitment of fast-twitch muscle fibers (Rønnestad et al., 2011; Hopker et al., 2017). Moreover, the observed increase in RPE from the first to second part of SINGLE aligns with previous studies investigating low-intensity training sessions (Rønnestad et al., 2011; Hopker et al., 2017; Talsnes et al., 2022), indicating an elevated perception of effort while maintaining the same external intensity over time.

The observed reduction in certain internal intensity measures (i.e., HR, Bla, and BG) from the first to second session of DOUBLE was notable. Specifically, we found significant interaction effects, indicating markedly lower levels of these intensity measures in the second session of DOUBLE compared to the second part of SINGLE. These findings are consistent with a recent study examining acute physiological responses to different organizational approaches of low-intensity training in cross-country skiers (Talsnes et al., 2022). In that study, reduced HR and Bla responses were observed in the second session of low-intensity training performed on the same day. The mechanisms underlying the reduced HR response in the second session of DOUBLE in our study may involve circadian variations (“time-of-day effects”) (Atkinson & Reilly, 1996; Chtourou & Souissi, 2012) and/or “preconditioning effects” from the first session (Kilduff et al., 2013). Additionally, the decreased Bla and BG levels from the first to second session of DOUBLE could be attributed to glycogen depletion and reduced CHO availability (Bartlett et al., 2015), despite the relatively short session duration and the provision of large amounts of exogenous CHO. However, these interpretations are not fully supported by the increased RER values observed in the second session of DOUBLE. Although the participants were instructed to replicate their dietary intake both the day before and the day constituting the experimental trial, no data on their nutritional intake or energy/CHO availability were included in the study. As such, the mechanisms driving the reduced physiological responses and particularly Bla and BG in the second session of DOUBLE remains speculative and should be investigated in future studies. Overall, these findings align with our hypotheses, suggesting a higher overall training stimulus when performing one long session compared to two shorter, time- and intensity-matched sessions of moderate-intensity training. Simultaneously, the lower physiological cost associated with the two shorter sessions indicates that this organization could allow for more accumulated time at this intensity in endurance athletes.

### Autonomic recovery

Performing one long moderate-intensity training session was further associated with higher supine HR during the 60-min recovery period following sessions compared to two shorter sessions. This outcome aligns with the expected higher acute physiological responses observed with SINGLE. Although measures of heart rate variability (HRV) were not included in the present study, previous research has demonstrated that both HR and HRV following endurance exercise are affected by exercise intensity, duration, and training status, and may therefore serve as indicators of autonomic recovery following endurance exercise (Cottin et al., 2004; Seiler et al., 2007). Considering this, the current findings suggest that performing a time- and intensity-matched long session of moderate-intensity training induces greater training stress (i.e., autonomic disturbance) and potentially different recovery demands compared to two shorter sessions. Therefore, differences in signal-to-stress ratios between different organization of moderate-intensity training in endurance athletes should further be investigated using measures of both autonomic and hormonal (e.g., blood biomarkers) disturbance.

### Measures of perceived training stress and recovery

Higher sRPE and internal training load (sRPE x duration) were evident in connection with SINGLE, indicating a higher perception of effort and most likely a higher overall training stimulus. This was further supported by increased levels of perceived fatigue and muscle soreness in the morning of the subsequent training day following SINGLE. These findings align with our hypotheses and reflect the higher overall physiological responses induced by performing one long session. Although no differences were found between SINGLE and DOUBLE in reported training quality (i.e., physical, technical, mental perspectives), lower motivation was reported before the second compared to first session of DOUBLE. This finding might be related to the participants’ lack of familiarity with performing “double-threshold training” and the laboratory-based nature of the design, which may differ from how this method would be implemented in a more ecologically valid setting.

### Practical applications and future research

Although we achieved high internal validity of the study protocol by employing a time- and intensity-matched laboratory design as a starting point, it differs somewhat from the actual use of the method in sports practice. One reported benefit of “double-threshold training” is to increase the overall volume of moderate-intensity training. Therefore, a logical next step would be to increase the duration of the two short sessions to achieve the same internal training load as one single session. In our case, an additional 10-min duration with the same sRPE for the two short sessions would be necessary to match the internal training load of the single session. Alternatively, two shorter sessions at the same internal intensity as one long session could be performed at a higher external intensity (i.e., more competition-relevant speed or power), thereby enhancing motor unit recruitment. Moreover, the ability to switch between exercise modes in sports that utilize different modes (e.g., triathlon and cross-country skiing) could further enhance the tolerance of moderate-intensity training due to variations or reductions in muscular and mechanical loading between sessions. Lastly, performing one longer session leads to a higher acute physiological response due to a duration-dependent “drift” in physiological measures, which may elicit a greater magnitude of molecular signaling (i.e., training stimulus) and influence subsequent adaptations differently.

## Conclusion

One long moderate-intensity training session was associated with a duration-dependent “drift” in acute physiological responses compared to two short time- and intensity-matched sessions. Simultaneously, the lower cost of the two shorter sessions indicates that such organization could allow for more accumulated time at this intensity. While future training intervention studies are required to investigate actual training effects, these findings serve as a starting point to better understand the pros and cons (i.e., different signal-to-stress ratios) of organizing moderate-intensity training as one long versus shorter sessions more frequently (e.g., as “double threshold training”) in endurance athletes.

## Data Availability

The raw data supporting the conclusions of this article will be made available by the authors, without undue reservation.
